# Development of a predictive model utilizing the neutrophil to lymphocyte ratio to predict neoadjuvant chemotherapy efficacy in early breast cancer patients

**DOI:** 10.1038/s41598-020-80037-2

**Published:** 2021-01-14

**Authors:** Jiujun Zhu, Dechuang Jiao, Yajie Zhao, Xuhui Guo, Yue Yang, Hui Xiao, Zhenzhen Liu

**Affiliations:** Department of Breast Cancer Center, Affiliated Cancer Hospital of Zhengzhou University, Henan Cancer Hospital, No. 127, Dongming Road, Zhengzhou, China

**Keywords:** Breast cancer, Cancer models, Tumour biomarkers

## Abstract

Neutrophils and lymphocytes are key regulators of breast cancer (BC) development and progression. Neutrophil to lymphocyte ratio (NLR) values have been found to offer clear prognostic utility when evaluating BC patients. In this study, we sought to determine whether BC patient baseline NLR values are correlated with pathological complete response (pCR) following neoadjuvant chemotherapy (NCT) treatment. In total, 346 BC patients underwent NCT at our hospital from January 1, 2014 to October 31, 2019, and data pertaining to these patients were retrospectively analyzed. Correlations between clinicopathological characteristics and pCR rates were assessed via multivariate logistic regression analyses. A predictive scoring model was used to gauge the likelihood of pCR based upon regression coefficient (β) values for each significant variable identified through these analyses. NLR cut-off values suitable for identifying patients likely to achieve pCR following NCT treatment were calculated using receiver operating characteristic (ROC) curves. All patients in the present study were females with a median age of 48 years old (range 22–77). An optimal NLR cut-off value of 1.695 was identified and was associated with respective sensitivity and specificity values of 63.6% and 45.5%. We found that higher NLR values were significantly associated with younger age, premenopausal status, and non-pCR status. Logistic regression analyses indicated that NLR, tumor size, hormone receptor (HR) status, and Ki-67 expression were all independent predictors of pCR. The area under the curve (AUC) for the resultant predictive scoring model was 0.705, and this model was assessed via K-fold cross-validation (k = 10) and bootstrapping validation, yielding respective AUC values of 0.68 and 0.694. Moreover, the incorporation of NLR into this predictive model incrementally improved its overall prognostic value relative to that of a model not incorporating NLR (AUC = 0.674). BC patients with a lower baseline NLR are more likely to exhibit pCR following NCT treatment, indicating that NLR may be a valuable biomarker for BC patient prognostic evaluation and treatment planning. Overall, our results demonstrate that this NLR-based predictive model can efficiently predict NCT efficacy in early BC patients with a high degree of accuracy.

## Introduction

Neoadjuvant chemotherapy (NCT) is commonly used to treat breast cancer (BC) patients with locally advanced or inflammatory disease in order to permit surgical treatment or to decrease tumor size and thereby better allow for breast-conserving surgery to be performed. Pathological complete response (pCR) rates following treatment are higher in BC patients with human epidermal growth factor receptor-2 positive (HER2+) or triple-negative (TN) relative to patients with HER2 negative (HER2−) or hormone receptor positive (HR+) disease. Some studies have suggested that pCR following NCT treatment can be used as a reliable indicator of patient disease-free survival (DFS) and overall survival (OS), particularly in patients with aggressive TNBC or HER2− BC^1^. As such, many studies have sought to identify clinicopathological variables associated with pCR in BC patients following NCT, with tumor size and grade being among the primary factors known to be predictive of this treatment outcome^2,3^. Additional studies have also demonstrated that particular genetic and molecular phenotypes can be used to predict NCT treatment outcomes^4^. However, the high costs associated with these genetic and molecular tests limit their utility in regular clinical practice. It is therefore vital that more readily accessible pathologic or laboratory biomarkers associated with NCT treatment outcomes be identified.

Inflammation is a key driver of cancer development and progression, and is closely linked to metastasis^5^. Neutrophils form an important component of the tumor-induced inflammatory response, whereas lymphocytes are most often related to anti-tumor immune responses such that neutrophil to lymphocyte ratio (NLR) values can reflect the dynamic balance between inflammatory and anti-inflammatory responses in cancer patients. As they serve as a readout for inflammatory status, NLR values have been employed to evaluate treatment efficacy and patient prognosis in cancer patients undergoing chemotherapy^6^. In BC, pretreatment NLR values have been found to be associated with patient prognosis. One meta-analysis found higher pretreatment NLR values to be associated with reduced DFS^7^. While BC tumors do not generate large quantities of neoantigens^8^, they are commonly subjected to lymphocyte infiltration, with variations in the degree of this infiltration across BC subtypes^9,10^. The presence of tumor-infiltrating lymphocytes (TILs) in BC patients is closely associated with pCR following treatment, with regulatory T cells (Tregs) and myeloid-derived suppressor cells (MDSCs) serving to suppress immune functionality and to reduce NCT efficacy in BC^11^. However, few studies to date have assessed whether pretreatment NLR values can predict prognosis and treatment efficacy in BC patients undergoing NCT treatment. While some studies have found a higher NLR to be associated with lower pCR rates in BC^6,12^, this finding is not universal and thus remains controversial^13,14^. The present study was therefore designed to explore the prognostic utility of NLR values as a tool for predicting NCT treatment outcomes in BC patients.

## Materials and methods

### Patients

In total, we retrospectively analyzed outcomes from 1435 primary BC patients that underwent NCT treatment at Henan Cancer Hospital from January 1, 2014 to October 31, 2019. Patients eligible for inclusion in this study were those that met the following criteria: (1) female; (2) had not undergone any invasive procedures or biopsies within one week prior to blood sample collection; (3) had a confirmed diagnosis of invasive BC as determined by core needle biopsy, with tumor estrogen receptor (ER), progesterone receptor (PR), HER2, and Ki-67 status being known; (4) had stage II-III BC, with T staging have been conducted via clinical examination, and with N staging having been conducted via needle aspiration biopsy or core needle biopsy in response to positive axillary lymph node (ALN) palpation or suspicious imaging findings (AJCC 7th edition); (5) patients did not have any contraindications precluding chemotherapy upon general examination; (6) underwent six standard NCT cycles; (7) surgery was conducted after NCT; and (8) breast and ALN pathological findings were available. Patients were excluded from this study if they: (1) were male; (2) had undergone invasive examinations within 1-week prior to blood sample collection; (3) had bilateral BC; (4) had evidence of active infection within the past week; (5) had inflammatory BC; (6) suffered from any chronic autoimmune or inflammatory disease or hematological disorders; or (7) underwent fewer than 6 NCT cycles. After screening patients using these criteria, 346 were included in our final analysis.

All HER2− patients received a standardized NCT regimen composed of six courses of TEC (75 mg/m^2^ docetaxel, 75 mg/m^2^ epirubicin, and 500 mg/m^2^ cyclophosphamide) every 3 weeks. Of the 140 patients in this study with HER2+ BC, 89 were administered tri-weekly TCH (docetaxel 75 mg/m^2^, carboplatin AUC 6, and trastuzumab 8 mg/kg loading dose, 6 mg/kg maintenance dose) treatment, while the remaining 51 HER2+ patients underwent TEC treatment. All patients underwent mastectomy or breast-conserving surgery and ALN dissection following NCT.

This study was approved by the Ethical Review Committee of the Affiliated Cancer Hospital of Zhengzhou University (No. 2019001). As this study had a retrospective design, the requirement for informed consent was waived by the hospital. We present this article in accordance with the Strengthening the Reporting of Observational Studies in Epidemiology (STROBE) Statement.

### Pathology

Tissue samples from BC patients were fixed using 10% formaldehyde, paraffin-embedded, and cut into serial 4 μm-thick sections that were subjected to routine hematoxylin and eosin (H&E) staining. Immunohistochemical (IHC) staining was then used to assess tumor ER, PR, HER2, and Ki-67 status. Tumors were considered to be ER or PR positive when at least 1% of tumor nuclei were positive for these hormone receptors, as per the corresponding American Society of Clinical Oncology/College of American Pathologists (ASCO/CAP) guideline^15^. Tumors were considered to be HER2-positive when they were scored either a 3+ via IHC or a 2+ with subsequent fluorescence in situ hybridization (FISH) confirmation, as per the corresponding ASCO/CAP HER2 guideline^16^. While Ki-67 cut-off criteria vary between centers, we defined a sample to be Ki-67-high if the proliferation index was > 30%^17^. Tumors were separated into four subtypes based upon HR and HER2 status: HR+HER2−, HR+HER2+, HR−HER2+, HR−HER2−. PCR was defined as the absence of any detectable tumor cell residue (ypT0ypN0) in primary breast tumors and axillary lymph node samples collected during surgery following NCT^1^.

### Blood data collection

Peripheral complete blood counts were performed at baseline in our hospital laboratory according to the standardized operative procedures. Blood data were collected by reviewing medical records. NLR was calculated as the ratio between the absolute neutrophil and lymphocyte counts.

### Statistical analysis

SPSS 17.0 and SAS 9.4 were used for all statistical analyses. Categorical variables were analyzed via chi-squared tests or Mann–Whitney *U* tests, with P < 0.05 as the significance threshold. Factors yielding a P-value < 0.1 in univariate analyses were incorporated into subsequent multivariate binary logistic regression analysis. Factors yielding a P-value < 0.05 in this multivariate analysis were considered to be independent related factors. The probability of pCR in this study was assessed by developing a predictive scoring model. This model was constructed by incorporating regression coefficient (β) values for each independent related variable identified in the above multivariate analyses. Numbers were rounded to the nearest whole number and were converted into scores, while negative numbers were added to a 1 or 2 in order to yield a positive number. The total risk score was then generated by summing together the individual scores for each variable. The NLR and risk score cut-off values were calculated based upon maximum Youden's index of the ROC curve. We additionally assessed the AUC, sensitivity, and specificity values associated with the chosen ROC curve cut-off values. The risk score discriminative abilities were assessed via K-fold cross-validation (k = 10) and bootstrapping validation.

## Result

### The relationship between NLR and patient clinicopathological parameters

**Figure 1:**
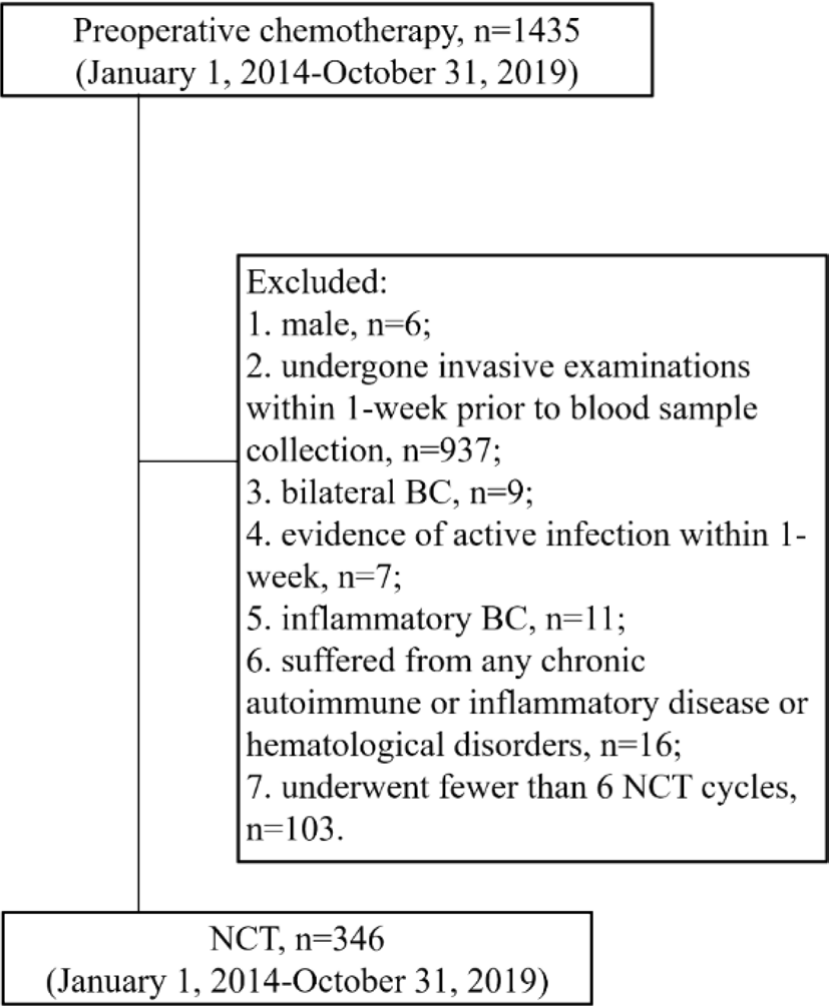
Patient flow diagram. *BC* breast cancer, *NCT* neoadjuvant chemotherapy.

In total, we retrospectively analyzed outcomes from 346 female BC patients (Fig. 1). These patients had a median age of 48 years-old (range 22–77), and 227 (66%) were premenopausal, 258 (75%) had T1-T2 tumors, 236 (68.2%) had HR+ tumors, 140 (40.5%) had Her2+ tumors, and 255 (73.7%) exhibited tumors with high Ki-67 expression (Table 1).

**Table 1:** Baseline patient characteristics (%).

Characteristics	Total	NLR		P
		Low	High	
**Age (years)**				
< 50	199 (57.5)	71 (35.7)	128 (64.3)	0.001
≥ 50	147 (42.5)	78 (53.1)	69 (46.9)	
**Menopausal**				
Premenopausal	227 (66)	84 (37)	143 (63)	0.001
Postmenopausal	117 (34)	65 (55.6)	52 (44.4)	
**Location**				
Upper outer	192 (55.7)	89 (46.4)	103 (53.6)	0.069
Lower outer	38 (11)	13 (34.2)	25 (65.8)	
Lower inner	25 (7.2)	9 (36)	16 (64)	
Upper inner	66 (19.1)	33 (50)	33 (50)	
Central	24 (7)	5 (20.8)	19 (79.2)	
**T**				
T1	29 (8.4)	14 (48.3)	15 (51.7)	0.585^#^
T2	229 (66.6)	95 (41.5)	134 (58.5)	
T3	62 (18)	25 (40.3)	37 (59.7)	
T4	24 (7)	13 (54.2)	11 (45.8)	
**N**				
N0	65 (18.8)	28 (43.1)	37 (56.9)	0.497^#^
N1	161 (46.5)	74 (46)	87 (54)	
N2	80 (23.1)	34 (42.5)	46 (57.5)	
N3	40 (11.6)	13 (32.5)	27 (67.5)	
**Hr**				
Negative	110 (31.8)	50 (45.5)	60 (54.5)	0.540
Positive	236 (68.2)	99 (41.9)	137 (58.1)	
**HER2**				
Negative	206 (59.5)	84 (40.8)	122 (59.2)	0.297
Positive	140 (40.5)	65 (46.4)	75 (53.6)	
**Ki-67**				
Low expression	91 (26.3)	41 (45.1)	50 (54.9)	0.655
High expression	255 (73.7)	108 (42.4)	147 (57.6)	
**Subtype**				
HR+HER2−	143 (41.3)	59 (41.3)	84 (58.7)	0.484
HR+HER2+	93 (26.9)	40 (43)	53 (57)	
HR−HER2+	47 (13.6)	25 (53.2)	22 (46.8)	
HR−HER2−	63 (18.2)	25 (39.7)	38 (60.3)	
**pCR**				
No	269 (77.7)	105 (39)	164 (61)	0.005
Yes	77 (22.3)	44 (57.1)	33(42.9)	
Total	346	149 (43.1)	197 (56.9)	

*HR* hormone receptor, *HER2* human epidermal growth factor receptor-2, *pCR* pathologic complete response, *NLR* neutrophil–lymphocyte ratio, + positive, − negative, # Mann–Whitney *U* test result.

**Figure 2:**
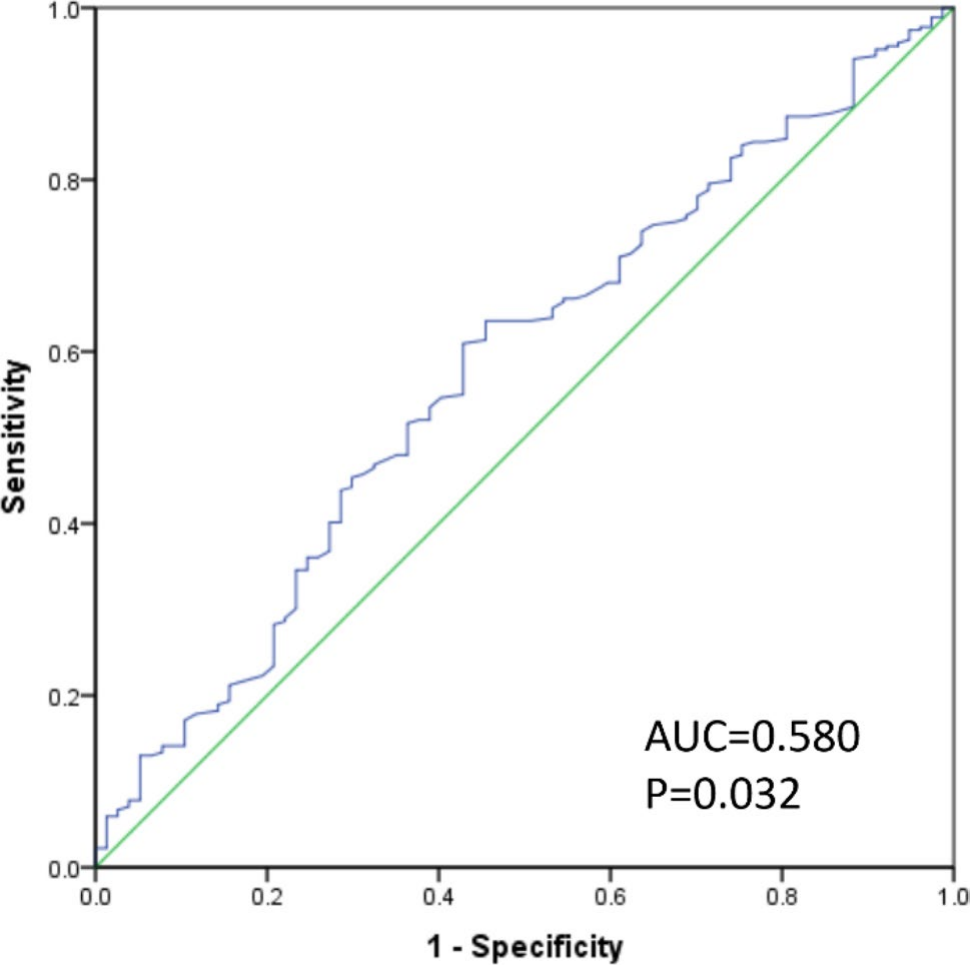
Receiver operating characteristic (ROC) and area under the curve (AUC) analyses pertaining to absolute neutrophil/lymphocyte ratio (NLR) at diagnosis.

NLR values were individually determined for each patient, and ranged from 0.45–9.36 (mean 2.01 ± 0.05; median 1.84; standard deviation 0.91). An ROC curve was constructed to examine the relationship between NLR and pCR following NCT. The AUC for this curve was 0.580 (95% CI 0.508–0.652; P = 0.032). An optimal cut-off value of 1.695 was selected according to the Youden's index, yielding respective sensitivity and specificity values of 63.6% and 45.5%. Of these patients, 149 (43.1%) were considered to have a low baseline NLR (< 1.695) and 197 (56.9%) were considered to have a high baseline NLR (≥ 1.695). We found that patients with a high NLR were more likely to younger (P = 0.001), premenopausal (P = 0.001), and to experience non-pCR (P = 0.005) (Table 1, Fig. 2).

### Univariate and multivariate analyses of the relationship between NLR and pCR

In total, 77 patients (22.3%) achieved pCR following NCT, with respective pCR rates of 16.8% and 29.5% in the high and low NLR groups (P = 0.005). Patients with small, HR−, HER2+, Ki-67-high tumors were more likely to achieve pCR following NCT (all P < 0.1). In addition, pCR was not found to be associated with patient age, menstrual status, tumor location, or lymph node status in these univariate analyses (all P > 0.1) (Table 2). A subsequent multivariate logistic regression analysis revealed NLR (OR 2.034, 95% CI 1.178–3.512, P = 0.011), tumor size, HR (OR 0.386, 95% CI 0.219–0.679, P = 0.001), and Ki-67 status (OR 2.318, 95% CI 1.104–4.868, P = 0.026) to all be independent predictors of patient pCR rates (Table 3).

**Table 2:** Correlations between patient clinicopathological features and pCR after NCT (%).

Characteristics	Total	pCR		P
		No	Yes	
**Age (years)**				
< 50	199 (57.5)	155 (77.9)	44 (22.1)	0.940
≥ 50	147 (42.5)	114 (77.6)	33 (22.4)	
**Menopausal**				
Premenopausal	227 (66)	175 (77.1)	52 (22.9)	0.745
Postmenopausal	117 (34)	92 (78.6)	25 (21.4)	
**Location**				
Upper outer	192 (55.7)	152 (79.2)	40 (20.8)	0.237
Lower outer	38 (11)	29 (76.3)	9 (23.7)	
Lower inner	25 (7.2)	20 (80)	5 (20)	
Upper inner	66 (19.1)	46 (69.7)	20 (30.3)	
Central	24 (7)	22 (91.7)	2 (8.3)	
**T**				
T1	29 (8.4)	17 (58.6)	12 (41.4)	0.037^#^
T2	229 (66.6)	180 (78.6)	49 (21.4)	
T3	62 (18)	53 (85.5)	9 (14.5)	
T4	24 (7)	19 (79.2)	5 (20.8)	
**N**				
N0	65 (18.8)	53 (81.5)	12 (18.5)	0.481^#^
N1	161 (46.5)	122 (75.8)	39 (24.2)	
N2	80 (23.1)	60 (75)	20 (25)	
N3	40 (11.6)	34 (85)	6 (15)	
Hr				
Negative	110 (31.8)	72 (65.5)	38 (34.5)	0.000
Positive	236 (68.2)	197 (83.5)	39 (16.5)	
**HER2**				
Negative	206 (59.5)	167 (81.1)	39 (18.9)	0.072
Positive	140 (40.5)	102 (72.9)	38 (27.1)	
**Ki-67**				
Low expression	91 (26.3)	80 (87.9)	11 (12.1)	0.007
High expression	255 (73.7)	189 (74.1)	66 (25.9)	
**Subtype**				
HR+HER2−	143 (41.3)	126 (88.1)	17 (11.9)	0.000
HR+HER2+	93 (26.9)	71 (76.3)	22 (23.7)	
HR−HER2+	47 (13.6)	31 (66)	16 (34)	
HR−HER2−	63 (18.2)	41 (65.1)	22 (34.9)	
**NLR**				
Low	149 (43.1)	105 (70.5)	44 (29.5)	0.005
High	197 (56.9)	164 (83.2)	33 (16.8)	
Total	346	269 (77.7)	77 (22.3)	

*HR* hormone receptor, *HER2* human epidermal growth factor receptor 2, *pCR* pathologic complete response, *NLR* neutrophil–lymphocyte ratio, + positive, − negative, # Mann–Whitney *U* test result.

**Table 3:** The results of multivariate logistic regression analysis.

Characteristics	Beta coefficient	P	OR	95% CI	
				Lower	Upper
T1		0.010			
T2	− 1.23	0.005	0.292	0.123	0.692
T3	− 1.836	0.001	0.160	0.053	0.479
T4	− 1.227	0.063	0.293	0.080	1.071
Hr positive	− 0.952	0.001	0.386	0.219	0.679
HER2 positive	0.405	0.146	1.499	0.869	2.587
Ki-67 high expression	0.841	0.026	2.318	1.104	4.868
NLR low	0.71	0.011	2.034	1.178	3.512
Constant	0.043	0.939	1.044		

*HR* hormone receptor, *HER2* human epidermal growth factor receptor 2, *NLR* neutrophil–lymphocyte ratio.

### Development of a predictive scoring model

We next sought to develop a predictive scoring model capable of estimating the odds of pCR following NCT in BC patients by utilizing the regression coefficient (β) values for each variable identified in the above analysis, with whole numbers being converted into corresponding scores. For example, β coefficients values for tumor of sizes T1, T2, T3, and T4 were 0, − 1.23, − 1.836, and − 1.227, respectively, and these values were rounded up and added to the number two to yield scores of 2, 1, 0, and 1, respectively. Total scores ranged from 0–5, and full details regarding this scoring system can be found in Table 4.

**Table 4:** The predictive scoring system.

Predictor	Prediction score points
**T**	
T1	2
T2	1
T3	0
T4	1
**Hr**	
Negative	1
Positive	0
**Ki-67**	
Low expression	0
High expression	1
**NLR**	
Low	1
High	0
Total	5

*HR* hormone receptor, *NLR* neutrophil–lymphocyte ratio.

### Predictive scoring model validation

**Figure 3:**
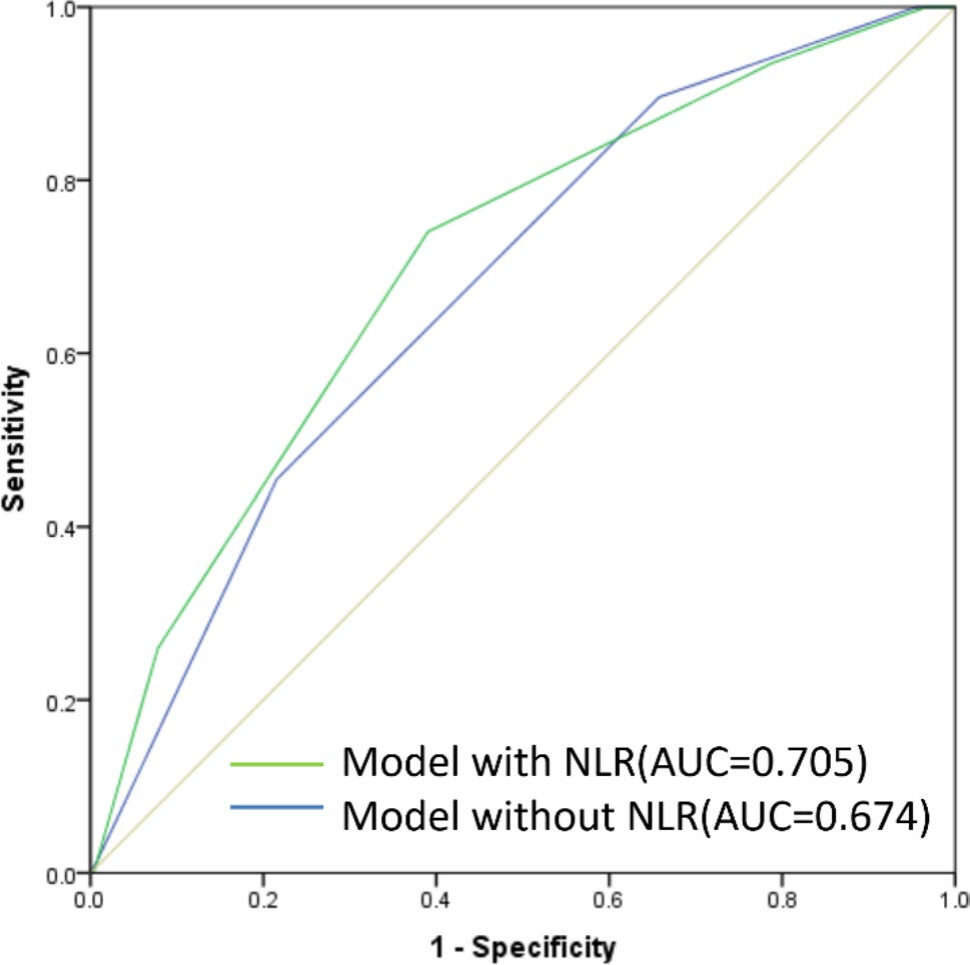
Receiver operating characteristic (ROC) and area under the curve (AUC) analyses pertaining to the predictive scoring model.

We next constructed an ROC curve evaluating the relationship between patient risk scores and pCR rates. This curve yielded an AUC of 0.705, whereas the AUC value for the model that did not include NLR was 0.674, consistent with the ability of our scoring system to readily differentiate between patients that would and would not achieve pCR following NCT (Fig. 3). A Hosmer–Lemeshow Goodness-of-Fit test confirmed that this model had a good fit (P = 0.891). We selected a score of 3 points as a cut-off value for estimating patient pCR status based upon the largest Youden's index value. Patients having a score of ≥ 3 were more likely to achieve pCR. This cut-off value yielded respective sensitivity, specificity, positive predictive, and negative predictive values of 74.0%, 61.0%, 35.2%, and 89.1% in this patient cohort (Table 5). In addition, the discriminative ability of these risk scores was assessed via K-fold cross-validation (k = 10) and bootstrapping validation, yielding respective AUC values of 0.68 and 0.694.

**Table 5:** The rate of pCR in the derivation cohort.

Prediction score points	pCR		Total
	No	Yes	
0	9 (100)	0	9 (2.6)
1	48 (90.6)	5 (9.4)	53 (15.3)
2	107 (87.7)	15 (12.3)	122 (35.3)
3	84 (69.4)	37 (30.6)	121 (35)
4	20 (50)	20 (50)	40 (11.6)
5	1 (100)	0	1 (0.3)
	269 (77.7)	77 (22.3)	346

*pCR* pathologic complete response.

## Discussion

Many studies to date have evaluated the predictive and prognostic potential of baseline NLR values as a means of assessing BC patients^18^. Herein, we expanded upon these prior studies by analyzing a cohort of 346 BC patients that had undergone NCT. Through these analyses, we determined that a low NLR was an independent predictor of pCR following NCT treatment.

We selected an NLR cut-off value of 1.695 in order to differentiate between patients that were and were not likely to achieve pCR following NCT. This cut-off was selected based upon ROC curve analyses. There is currently no unified consensus regarding optimal NLR cut-off values for the evaluation of BC patients. Prior studies of BC patients undergoing NCT have utilized NLR cut-off values ranging from 1.7–3.33, and a recent meta-analysis incorporating 39 studies and 17,079 total BC patients undergoing adjuvant chemotherapy similarly confirmed this lack of cut-off consistency (range 1.7–4.0)^7,19,20^. The cut-off value selected in the present study was lower than the values reported in prior studies. This may be due to site-specific differences or may suggest that inclusion/exclusion criteria in our study were more stringent. No patients that had undergone any invasive treatments or evaluations prior to blood sample collection were included in our study, as our inclusion/exclusion criteria were more stringent than prior studies.

When we analyzed the relationship between NLR and other clinicopathological characteristics in these BC patients, we found that younger patients and premenopausal patients were more likely to have higher NLR values. This was consistent with the results of a prior study conducted in Mexico which determined that patients with an NLR > 2 tended to be younger^13^. Together, these results indicate that NLR values may be associated with differential effects in younger and older patients, although further research will be necessary to confirm these findings.

Multiple studies to date have reported on the prognostic value of NLR as a means of assessing outcomes in patients undergoing NCT^6,21,22^. In a 2019 meta-analysis of 11 studies incorporating 2107 BC patients, lower NLR values were found to be associated with significantly better NCT responses^23^. However, a large single study incorporating 1519 BC patients did not detect any relationship between NLR and pCR rates^13^. As such, the prognostic relevance of NLR values remains controversial in BC patients undergoing NCT. In the present study, 77 patients (22.3%) achieved pCR following NCT, with significantly better pCR rates in the low NLR group relative to the high NLR group (29.5% vs. 16.8%). In a multivariate analysis, baseline NLR, tumor size, HR status, and Ki-67 status were all confirmed to be independent predictors of pCR. There may be two primary factors explaining the inconsistency between our findings and those of certain prior studies. For one, we may have selected patients more rigorously by specifically excluding individuals that had undergone invasive procedures such as core needle biopsy prior to blood sample collection. Second, NCT regimens in the abovementioned prior study were not uniform, whereas relatively uniform chemotherapy regimens were applied to all patents in our study. We excluded patients that had undergone alternative treatment regimens or an inadequate number of NCT cycles. Based on our findings and those of prior meta-analyses, we can conclude that NLR values may serve as valuable and effective predictors of pCR following NCT treatment in BC patients.

Further research is required in order to understand why patients with low NLR values are more sensitive to NCT treatment. BC development is closely associated with inflammation and with the overall immune status of affected patients, and tumor cells are capable of releasing many non-specific inflammatory cytokines and other factors that can activate or recruit other inflammatory cells capable of promoting angiogenesis and disease progression^24^. Inflammatory responses are characterized by the recruitment and activation of neutrophils, lymphocytes, and other cell types, and by the production of C-reactive protein. Neutrophils in particular can secrete inflammatory cytokines and can promote tumor metastasis and angiogenesis^25^. Reduced lymphocyte counts may be indicative of impaired anti-tumor immune responses, enabling cancer cells to more readily grow and spread^26^. As such, NLR values serve as a dynamic indicator of the overall immune status in a given patient, making them of key relevance in the field of oncology. Oncogenesis and cancer progression result in a loss of homeostatic balance between pro- and anti-inflammatory responses, such that lymphocyte counts tend to decrease and neutrophil/platelet counts tend to rise, leading to higher NLR and PLR values^24^. Ozyalvacli et al. assessed NLR distributions in BC patients and in 50 controls with benign breast diseases, confirming that BC patients exhibited significantly higher average NLR values^27^. Three other studies have also suggested that the risk of BC increased with rising NLR levels^28–30^. In addition, cancer stem cells make up a small subset of overall tumor cells, and possess the ability to self-renew and differentiate indefinitely. These stem cells are important mediators of tumor aggression, chemoresistance, and metastasis^31^. Tumor-associated neutrophils produce large quantities of bone morphogenic protein (BMP)-2 and TGF-β2, which confer stem-like properties to these cancer cells^32^.

Following a multivariate analysis identifying independent predictors of pCR, we developed a scoring system capable of more reliably predicting pCR rates in BC patients undergoing NCT. Several prior studies have reported similar models incorporating variables such as tumor-infiltrating lymphocyte scores or tumor cellularity on day 15 of treatment, with these models having been found to predict patient outcomes after completion of neoadjuvant anti-HER2-based therapy^33^. Our model, however, is more accessible than these prior models, as it incorporates NLR values which can be readily obtained. Our model yielded an AUC of 0.705, indicating that it can effectively differentiate between patients that do and do not achieve pCR. Moreover, the addition of NLR also provided an incremental improvement in the prognostic value of our model relative to one not incorporating NLR (AUC = 0.674). Internal verification of this model via K-fold cross-validation (k = 10) and bootstrapping validation yielded respective AUC values of 0.68 and 0.694, indicating it to be stable and satisfactory. As such, our model represents a valuable clinical tool for the priori assessment of which BC patients are most likely to achieve pCR following NCT. By accurately predicting the odds of pCR prior to surgery, clinicians can more effectively weigh the relative costs and benefits of such treatment so as to ensure that they act in the best interest of the patient.

There are multiple strengths to this study. For one, we provided a detailed overview of the NCT regimens used to treat patients, consistent with international recommendations. Second, a unified NCT regimen was used for all patients, thus precluding the possibility that regimen-specific differences may have influenced patient pCR rates. Third, we only enrolled patients that had not undergone any invasive procedures prior to peripheral blood collection. However, there are also certain limitations to this study. Specifically, this was a retrospective study without any external validation and without any assessment of patient survival outcomes. Future studies will thus be needed to confirm and expand upon our findings.

In conclusion, our findings indicate that NLR values can be reliably used to predict BC patient responses to NCT treatment. Importantly, we generated a risk scoring model incorporating several easily-measured baseline clinicopathological parameters that can effectively stratify patients based upon their likelihood of achieving pCR. Patients with higher scores derived from this model were more likely to achieve pCR following NCT treatment. Future large-scale prospective studies, however, will be needed to confirm our findings.
